# Positive Effects of Laser Acupuncture in Methamphetamine Users Undergoing Group Cognitive Behavioral Therapy: A Pilot Study

**DOI:** 10.1155/2021/5514873

**Published:** 2021-05-22

**Authors:** Yi-Hsien Shiao, Yi-Chih Chen, Yuan-Chieh Yeh, Tse-Hung Huang

**Affiliations:** ^1^Department of Traditional Chinese Medicine, Chang Gung Memorial Hospital, Keelung Medical Center, Keelung, Taiwan; ^2^Department of Psychiatry, Chang Gung Memorial Hospital, Keelung Medical Center, Keelung, Taiwan; ^3^Program in Molecular Medicine, School of Life Sciences, National Yang Ming University, Taipei, Taiwan; ^4^Graduate Institute of Clinical Medicine Sciences, College of Medicine, Chang Gung University, Taoyuan, Taiwan

## Abstract

**Background:**

Methamphetamine (MA) addiction has become a crucial public health concern because of its adverse consequences to individuals and the society.

**Objective:**

To investigate the clinical efficacy of laser acupuncture combined with group cognitive behavioral therapy for MA addiction treatment.

**Materials and Methods:**

MA users who participated in group cognitive behavioral therapy and met the inclusion criteria were referred from psychiatrists to participate. The participants received laser acupuncture treatment once a week for 2 months (total eight treatments) on selected acupoints (PC6, HT7, LI4, ST36, SP6, and LR3). Laboratory assessment included urinalysis for MA and liver function tests aspartate aminotransferase, alanine aminotransferase, and *γ*-glutamyltransferase (AST, ALT, and *γ*-GT), whereas the objective assessment included visual analog scale (VAS) for MA craving and refusal and Pittsburgh sleep quality index (PSQI), Beck Anxiety Inventory (BAI), and Beck Depression Inventory (BDI) questionnaires. All data were collected before and at 1 and 2 months after treatment. Cognitive behavioral therapy completion rate and rate of relapse to MA use were also determined.

**Result:**

Fifteen participants were enrolled, of whom seven completed the trial. Urinalysis for MA revealed a decrease in drug use from 57.1% to 28.6%. Compared with those before treatment, PSQI scores were significantly lower at 1 and 2 months after treatment (−3.73 and −4.10, respectively; both *p* < 0.001), and so were BDI scores (−5.64 and −8.17, respectively; *p*=0.01 and 0.001, respectively). However, no significant difference was observed in the liver function test, VAS of craving and refusal, and BAI results. A slight improvement in the motivation for drug abstinence and anxiety was observed during the treatment course. Participants reported no adverse events.

**Conclusion:**

Laser acupuncture combined with group cognitive behavioral therapy may improve sleep quality, alleviate depression, and reduce MA use. Additional large-scale studies confirming the effectiveness of this modality are warranted.

## 1. Introduction

Methamphetamine (MA) has become one of the most commonly abused drugs in the world. According to data from the United Nations Office on Drugs and Crime, global prevalence of MA use reached 34,160,000 (0.7%) people in 2016 [1]. The quarterly report on drug use from Taiwan High Prosecutors Office in 2019 revealed that MA (including MA and amphetamine) was the second common drug abused substance; it was used by 35,304 people, corresponding to approximately 60% of all drug abusers in Taiwan [2]; however, these numbers might be underestimated. MA is mostly used by young people (aged 20–29 years) [3]. Withdrawal symptoms in abstinent MA-dependent individuals include depression, somnolence, anxiety, irritability, inability to concentrate, psychomotor slowing, increased appetite, and paranoia [4]. Therefore, it can potentially cause serious economic, social, and family problems.

Current evidence regarding the treatment options for MA addiction is insufficient and inconsistent [5]. Outpatient group cognitive behavioral therapy (CBT) is the standard treatment for MA abuse and dependence [4]. Although group CBT engenders some benefits to reduce MA use [6, 7], one study indicated that evidence on the effectiveness of group CBT in MA use disorders is insufficient [8]. In Taiwan, MA is classified as a class 2 drug according to its drug-prevention laws. Unlike heroin, there was no proved effective drug such as methadone for MA withdrawal treatment. Therefore, MA users if met deferred prosecution who received diversion program may participate in an outpatient relapse prevention program, which employs a CBT model, once a week over the course of 12 weeks [9]. The program, conducted by both a psychiatrist and a psychotherapist emphasize skills acquisition that can aid in reducing drug use and resolving personal, social, and environmental barriers [10]. However, our previous study revealed that 69% of MA users had at least one positive urine result during the whole relapse prevention program [11]. Thus, an alternative therapy must be urgently developed to address MA abuse.

Acupuncture is an alternative treatment for drug abuse. In particular, manual or auricular acupuncture have been applied in opioid dependence treatment for a long time [12, 13]. Electroacupuncture may also alleviate the symptoms of MA addiction such as psychosis, anxiety, and depression during abstinence [14]. Acupuncture clearly has a role to play drug-dependence treatment.

However, several comorbidities are associated with MA use, such as hepatitis B virus (HBV), hepatitis C virus (HCV), or human immunodeficiency virus (HIV) infection [15], which potentially exposes acupuncture physicians and medical practitioners to more risk of bloodborne infections. In addition, MA users may avoid traditional needle acupuncture because of their characteristic anxiety and increased sensitivity to pain [16]. Moreover, a conventional manual acupuncture session of 30 minutes to 1 hour may not be suitable for MA users who exhibit agitation. Therefore, a modified acupuncture procedure should be established to make treatment practicable.

The advantages of laser acupuncture (LA) include its safety, painlessness, limited time use, and higher acceptability, making it suitable for treatment of drug users. However, no study has investigated its therapeutic effect in the treatment of MA addiction. Therefore, this study evaluated the effectiveness of LA combined with group CBT for MA addiction treatment.

## 2. Materials and Methods

### 2.1. Study Design and Participants

In this pilot study, LA treatment was combined with standard group CBT, and it was conducted at Chang Gung Memorial Hospital, Keelung, Taiwan. Because this study was to be conducted at a single center including few MA users, the trial was designed to recruit 15 participants. A pilot trial sample size per treatment arm of 15 is considered medium in terms of standardized effect sizes [17]. The clinical trial was approved by the Human Committee of Chang Gung Memorial Hospital (Institutional Review Board of Chang Gung Medical Foundation, permit no. 201800431A3) and sponsored by the Ministry of Health and Welfare in Taiwan (project no. MOHW107-CMAP-M-114-122115). The registration of clinical trial in Clinicaltrial.com was NCT04789772. Informed consent was obtained from each participant.

We included patients who were aged ≥20 years, with full understanding of the content of the study and who signed a consent form; had received a diagnosis of MA use disorder by a psychiatrist according to the Diagnostic and Statistical Manual of Mental Disorders, Fifth Edition (DSM-V); and were group CBT participants. By contrast, we excluded patients who had received manual acupuncture or LA treatment in the preceding month; had cancer, stroke, myocardial infarction, or major trauma; demonstrated obvious suicide intention; were pregnant; and were HIV-positive.

### 2.2. Intervention

**Figure 1:**
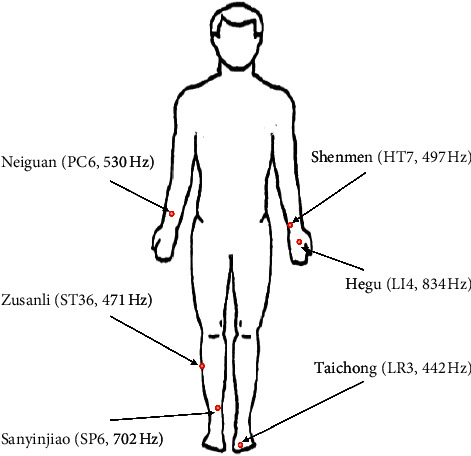
Selected acupoints and LA frequencies.

Consider MA users as the invulnerable group with socially isolated characteristics; the study only designed to set one group of LA treatment to evaluate the immediate effect and set shorter duration of 8 weeks course to lower dropout rate [18]. On the basis of traditional Chinese medicine theory and a literature review, six acupoints were selected for use in participants: PC6 (Neiguan), HT7 (Shenmen), LI4 (Hegu), ST36 (Zusanli), SP6 (Sanyinjiao), and LR3 (Taichong) (Figure 1). LA was performed once a week for 2 months for a total of eight sessions. Participants were asked to relax in a sitting position. Each of the selected acupoints was stimulated at 6 J by using the gallium aluminum arsenide LaserPan [19] (maximal power, 150 mW; wavelength, 810 nm; area of probe, 0.13 cm^2^; power density, 1.19 W/cm^2^; pulsed wave) (RJ-Laser, Reimers and Janssen GmbH, Waldkirch, Germany) with the following Reininger meridian frequency modes: Neiguan (PC6, pericardium; 530 Hz), Shenmen (HT7, heart; 497 Hz), Hegu (LI4, lung; 834 Hz), Zusanli (ST36, stomach; 471 Hz), Sanyinjiao (SP6, spleen; 702 Hz), Taichong (LR3, liver; 442 Hz). The laser acupuncture has a maximum power of 150 mW with pulsed laser output. It takes 80 sec of each of acupoint (6 J/150 mW*∗*2 = 80 s). All the LA procedures were performed by well-trained licensed traditional Chinese medicine physicians.

### 2.3. Outcome Measurements

Laboratory assessment included subjective and objective measurements. Considering the common comorbidity of hepatitis among MA users, urinalysis and liver function tests (AST, ALT, and *γ*-GT) were performed as the primary outcome measurements. Furthermore, to investigate secondary outcomes, including the effect on drug abstinence motivation, sleep quality, and MA-related mood disorders, visual analog scale (VAS) for MA craving and refusal and Pittsburgh sleep quality index (PSQI), Beck Anxiety Inventory (BAI), and Beck Depression Inventory (BDI) questionnaires were used. To evaluate the therapeutic duration, we noted the completion rate of the 12-week relapse prevention program of group CBT and rate of relapse to MA use after a 2-month follow-up. For this study, relapse was defined as a positive urine result for MA, and dropout was defined as being absent from three or more LA or CBT sessions.

### 2.4. Statistical Analysis

Statistical analysis was performed using SPSS (version 23.0 for Windows; IBM Corp., Armonk, NY, USA). A *p* value of <0.05 was considered statistically significant. Generalized estimating equation analysis was used to assess improvement rates for urinary MA, AST, ALT, and *γ*-GT levels as well as VAS for MA craving and refusal, PSQI, BAI, and BDI scores.

## 3. Results

Between January 2018 and December 2018, 15 individuals were referred from the psychiatry department, all of whom met the inclusion criteria. Of them, 5 (33.3%) were married. Their average education level was 11.5 years. The proportion of male users (73.3%) was more than that of female users (26.6%). The average age at first MA use was 26.7-year-old with average weekly use frequency of 3.27 times. The average MA use duration was 4.2 years. Four (26.6%) participants used illicit drugs other than MA, including heroin. Moreover, two (13.3%) participants had HBV infection, six (40%) had HCV infection, and four (26.6%) had psychiatric disorders. Before undergoing LA combined with group CBT, eight (53.3%) participants had a positive urinalysis results for MA. Subjects called MA users were defined as diagnosis by psychiatrists after arresting. After they read and signed the informed consent form, the investigators arranged the first urine test before the treatment of the study as baseline. Therefore, some of MA users still use MA privately and others were not.

**Figure 2:**
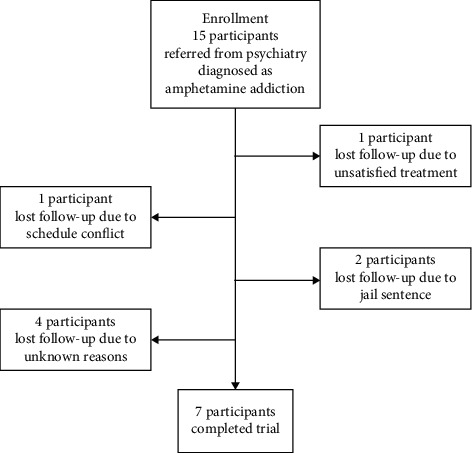
Flowchart of the study.

Seven (46.7%) participants fully completed this trial (Table 1). Participant loss was attributable to dissatisfaction with the treatment (*n* = 1), scheduling conflict (*n* = 2), the imposition of jail sentences (*n* = 2), and refusal of follow-up treatment for unknown reasons (*n* = 4; Figure 2).

### 3.1. LA Combined with Group CBT Reduced Rate of Relapse to MA Use and Improved 12-Week Relapse Prevention Program Completion Rate

Laboratory assessment results revealed that LA combined with group CBT reduced MA use from 57.1% to 28.6% compared with those who dropped out of the trial (from 50% to 62.5%) at 2 months after treatment (Table 1). Moreover, the 12-week relapse prevention program completion rate was higher in participants who completed this study (85.7%) than in those who did not (50%; Table 1).

### 3.2. LA Combined with Group CBT Improved Sleep Quality and Alleviated Depression

**Figure 3:**
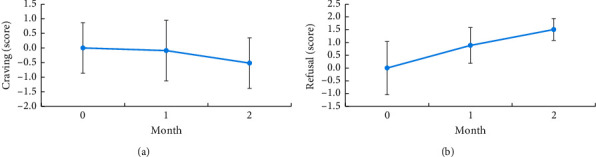
Line chart of mean change difference in scores on VAS of craving (a) and refusal (b) at 1 and 2 months of treatment. ^*∗*^*P* < 0.05 (significant), ^*∗∗*^*P* < 0.01 (highly significant), and ^*∗∗∗*^*P* < 0.001 (extremely significant).

**Figure 4:**
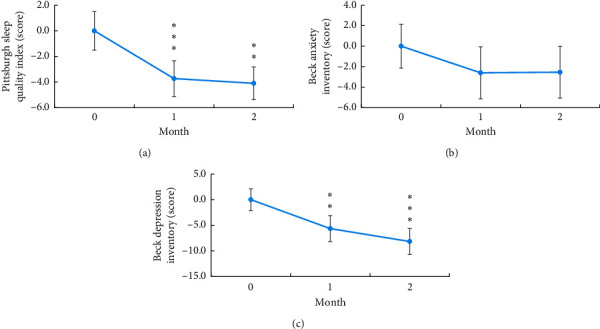
Line chart of mean change difference in scores on PSQI (a), BDI (b), and BAI (c) at 1 and 2 months after treatment. ^*∗*^*P* < 0.05 (significant), ^*∗∗*^*P* < 0.01 (highly significant), and ^*∗∗∗*^*P* < 0.001 (extremely significant).

LA combined with group CBT significantly reduced the PSQI scores at 1 and 2 months after treatment (−3.73 and −4.10, respectively; both *p* < 0.001) and the BDI scores at 1 and 2 months after treatment (−5.64 and −8.17, respectively; *p*=0.01 and 0.001, respectively). Although no significant difference was observed for scores on VAS for MA craving and refusal and BAI, a declining trend was noted in scores on VAS for craving and BAI, whereas a mild elevation was noted in scores on VAS for refusal (Table 2 and Figures 3 and 4).

### 3.3. LA Combined with Group CBT Does Not Reduce Liver Function

**Figure 5:**
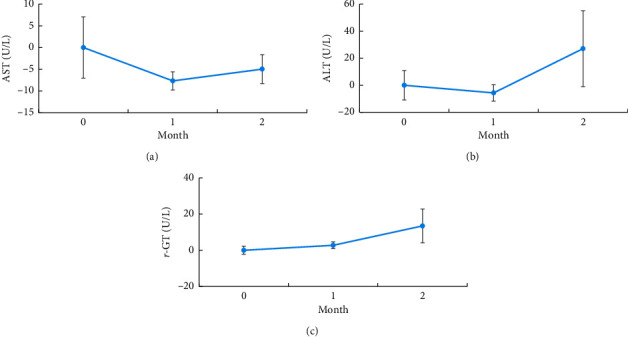
Line chart of mean change difference in AST (a), ALT (b), and *γ*-GT (c) levels at 1 and 2 months after treatment. ^*∗*^*P* < 0.05 (significant), ^*∗∗*^*P* < 0.01 (highly significant), and ^*∗∗∗*^*P* < 0.001 (extremely significant).

Laboratory assessment revealed no significant difference in AST, ALT, and *γ*-GT levels before and after treatment. However, a declining trend was noted in AST levels, whereas a mild elevation was noted in ALT and *γ*-GT levels, but within the normal range (Table 3 and Figure 5).

## 4. Discussion

Manual acupuncture has been used for drug detoxification. Acupuncture suppressed MA self-administration behavior through the regulation of the GABA system when performed on the HT7 acupoint of rats [20]. Russell et al. conducted a pilot study at the Consortium Treatment Center by using auricular acupuncture, which revealed that patients receiving acupuncture had significantly higher cumulative probability of remaining in treatment than did patients without acupuncture treatment, particularly patients with MA addiction [21]. LA has been used for more than 40 years in various aspects of human disease [22]. With its advantages of being noninvasive and having fewer adverse effects, LA has been widely used as a safer and pain-free alternative to manual acupuncture.

Depending on previous studies, the most common used acupoints for opioid detoxification are Neiguan (PC6), Zusanli (ST36), Sanyinjiao (SP6), Shenmen (HT7), Hegu (LI4) [23]. PC6 was used to alleviate chest congestion, nausea, and dizziness [24]. ST36 was used to alleviate abdominal discomforts [25] and depressive psychosis. SP6 was used to alleviate irregular menstruation and insomnia. HT7 was used to alleviate insomnia and palpitation [26]. LI4 was used to deal with disease of head and face, such as headache, dizziness, or toothache. Besides, the emotional dysregulation was related to dysfunction “liver meridian” of traditional Chinese medicine theory. Therefore, LR3 with function of depression release was selected as our treatment acupoint as well.

Our study revealed positive effects of LA combined with group CBT in treating MA addiction, including decreased MA use, improved completion rate of the 12-week relapse prevention program of group CBT, enhanced sleep quality, and alleviated depression. Although bias might have been involved in the lower reuse rate of MA among those who completed the study protocol because they might pay more attention to their health, some benefits were observed in LA treatment in terms of MA abstinence. Robust evidence suggests downregulated presynaptic and postsynaptic dopamine function in stimulant addiction through cocaine, amphetamine, or MA use [27]. In an animal study, LA application to acupoint HT7 could improve dopaminergic function of Parkinson disease [28]. No direct evidence has revealed the mechanism of LA in alleviating MA craving; however, we believe it might be related to dopaminergic receptor regulation by the low-level laser energy.

With regard to the effect of LA on the mood disorders in MA user, the PSQI and BDI scores revealed significant decreases. In a study, LA improved the sleep quality of patients with chronic insomnia [29]. In addition, acupuncture, including manual acupuncture, electroacupuncture, and LA, may be a generally beneficial, well-tolerated, and safe monotherapy for depression [30]. The antidepressant mechanism of LA presumably involves wider posterior default mode network modulation at the parietotemporal limbic cortices [31]. LA may be beneficial to improve the sleep quality and depressive status of MA users [29]. Although our BAI scores revealed no significant differences between pretreatment and posttreatment outcomes, a clearly declining level of anxiety was noted. These results are consistent with results of the trial by Zeng [14]: electroacupuncture effectively improved psychosis, anxiety, and depression symptoms during abstinence in patients with MA addiction. Based on previous analysis, the most common used acupoints to deal with anxiety are points belong to “bladder meridian” and “gallbladder meridian” [32]. Further clinical trial study should enroll these acupoints to make anxiety better.

MA can be ingested orally, inhaled, or injected. The sharing or reusing of needles among injecting drug users increases the chance of bloodborne infection. Global prevalence of HCV infection among injecting drug users is at least 50% in 49 countries or territories [33] and local prevalence is 41% in Taiwan [34]. Furthermore, HBV is still a commonly transmitted disease in Taiwan despite a successful vaccination program [35]. According to TCM theory, some acupoint such as LR3 can be used to balance liver meridian energy and treat liver diseases. One study points out that ST36 and LR3 may be effective in treating liver injury induced by carbon tetrachloride in rats [36]. Besides, one study showed that laser diode treatment applied to animal on Ganshu point (BL18) can repair live damage [37]. To prove the concept and investigate the effects of LA on hepatitis, regardless of vertical transmission or infection through needle use, we collected liver function data, including AST, ALT, and *γ*-GT from participants. The result showed that no significant difference before and after treatment. The average ALT and *γ*-GT did increase slightly after treatment as a result of coincidental HCV flare-ups in two participants during the course of treatment. Although, the result cannot prove our concept, there was no harmful effect on subjects under LA short-term treatment as last.

In a study, 69% of MA users had at least one positive urine result throughout a whole 12-week relapse prevention program [11], and 61% of the MA users relapsed to MA use within 1 year after treatment [38]. MA users struggle to completely stop using MA even when undergoing group CBT [11]. We also observed that those in our study who received the LA combined group CBT had a lower dropout rate (14.2%) compared with another study (40.5%) [11]. Although some subjects also used illicit drugs other than MA, they merely took up a small part. Briefly, the majority of illicit drug use was MA according to our data. Thus, it may cause a neglectable effect on complete rate of CBT course. Because of our current small sample size, drawing definite conclusions is difficult. Nevertheless, LA combined with group CBT may be a new effective modality for enabling MA abstinence.

People misuse MA to boost performance at work or in sexual relationships, to promote a sense of social belonging, and to help manage stress [39]. In addition, substance use is often associated with a personality disorder. In a recent Norwegian study, 46% patients with substance use disorder had at least one personality disorder (16% antisocial, male only; 13% borderline; and 8% paranoid, avoidant, and obsessive-compulsive) [40]. Among illicit drug users, antisocial and borderline personality disorders are the most common [40]. Therefore, MA use leads drug abusers to become more isolated, disconnected from their social circle, and unwilling to remain in hospital. To increase the treatment return rate, the LA sessions were coordinated with the group CBT schedule as one treatment session per week. However, half of all participants were lost during follow-up, with reasons including scheduling conflicts, prison sentences, and dissatisfaction with treatment. Furthermore, some MA users left incorrect contact phone numbers, which increased the difficulty to obtain an adequate sample size for a stronger conclusion.

### 4.1. Limitations

This study has several limitations. Because the group CBT program was conducted at an outpatient department, not at a rehabilitation center, it was prone to loss of follow-up and a decreased treatment return rate. Thus, this study had a small sample size due to single center study. Second, because drug users belong to a vulnerable group, no control group receiving only LA was set up to compare results. Third, the protocol was observed for only 2 months. The recording of the long-term posttreatment effect is required for increased accuracy. Because of the aforementioned factors, a multicenter, large-scale, long-term clinical trial with a control group is warranted for further evaluation in the future.

## 5. Conclusion

LA combined with group CBT may reduce relapse to MA use, improve group CBT completion rate and sleep quality, and alleviate depression during a 2-month follow-up period. Providing an appropriate clinical relapse prevention program for MA users remains a challenge. To the best of our knowledge, this is the first study to evaluate the effectiveness of LA combined with group CBT on MA detoxification. Our evidence indicated that multitherapy for MA abstinence could be better than group CBT alone. Additional large-scale studies confirming these therapeutic effects are warranted.

## Figures and Tables

**Table 1:** Participant characteristics users.

	Mean or *N*	SD or %
Demographic variables		
Age	42	10.24
Gender (female vs. male)	4/11	26.6/73.3
Marital status (married)	5	33.3
Level of education (years)	11.5	0.73

MA use patterns		
Age of onset of MA use	26.7	2.45
Weekly use of MA (times)	3.27	1.19
Duration use of MA (years)	4.2	0.77
Polysubstance use (yes vs. no)	4	26.6

Comorbidities		
HBV carrier	2	13.3
HCV carrier	6	40
Psychiatric disorder	4	26.6

LA + CBT treatment		
Completion	7	46.7
Dropout	8	53.3

CBT program		
LA + CBT (*N* = 7)		
Completion	6	85.7
Dropout	1	14.3
LA dropout (*N* = 8)		
Completion	4	50
Dropout	4	50

MA urine positive result		
Before treatment (*N* = 15)	8	53.3
LA + CBT (*N* = 7)		
Baseline	4	57.1
1-month follow	0	0
2-month follow	2	28.6
LA dropout (*N* = 8)		
Baseline	4	50
2-month follow	5	62.5

**Table 2:** Generalized estimating equation analysis for scores on VAS for craving and refusal, PSQI, BAI, and BDI.

		*β*	Standard error	*P*
VAS for craving	1^st^ month vs. baseline	−0.1	0.7	0.90
	2^nd^ month vs. baseline	−0.5	0.7	0.48

VAS for refusal	1^st^ month vs. baseline	0.9	1.1	0.40
	2^nd^ month vs. baseline	1.5	1.0	0.14

PSQI	1^st^ month vs. baseline	−3.7	0.7	<0.001^*∗∗∗*^
	2^nd^ month vs. baseline	−4.1	0.7	<0.001^*∗∗*^

BDI	1^st^ month vs. baseline	−5.6	2.2	0.01^*∗∗*^
	2^nd^ month vs. baseline	−8.2	2.4	<0.001^*∗∗∗*^

BAI	1^st^ month vs. baseline	−2.6	1.3	0.05
	2^nd^ month vs. baseline	−2.5	2.1	0.22

VAS, visual analog scale; PSQI, Pittsburgh sleep quality index; BAI, Beck Anxiety Inventory; BDI, Beck Depression Inventory. ^*∗*^*p* < 0.05; ^*∗∗*^*p* < 0.01; ^*∗∗∗*^*p* < 0.001.

**Table 3:** Generalized estimating equation analysis of urine MA and morphine levels and AST, ALT, and *γ*-GT levels.

		*β*	Standard error	*P*
AST	1^st^ month vs. baseline	−7.7	5.6	0.17
	2^nd^ month vs. baseline	−5.0	5.0	0.33

ALT	1^st^ month vs. baseline	−5.6	7.1	0.43
	2^nd^ month vs. baseline	27.1	22.4	0.23

*γ*-GT	1^st^ month vs. baseline	2.8	2.2	0.20
	2^nd^ month vs. baseline	13.5	9.5	0.16

^*∗*^ *P* < 0.05; ^*∗∗*^*P* < 0.01; ^*∗∗∗*^*P* < 0.001.

## Data Availability

The data used to support the findings of this study are available from the corresponding author upon request.
